# Genetic Background and Expression of the New *qepA4* Gene Variant Recovered in Clinical TEM-1- and CMY-2-Producing *Escherichia coli*

**DOI:** 10.3389/fmicb.2017.01899

**Published:** 2017-10-09

**Authors:** Vera Manageiro, David Félix, Daniela Jones-Dias, Daniel A. Sampaio, Luís Vieira, Luísa Sancho, Eugénia Ferreira, Manuela Caniça

**Affiliations:** ^1^National Reference Laboratory of Antibiotic Resistances and Healthcare Associated Infections, Department of Infectious Diseases, National Institute of Health Doutor Ricardo Jorge, Lisbon, Portugal; ^2^Centre for the Studies of Animal Science, Institute of Agrarian and Agri-Food Sciences and Technologies, University of Porto, Oporto, Portugal; ^3^Innovation and Technology Unit, Human Genetics Department, National Institute of Health Doutor Ricardo Jorge, Lisbon, Portugal; ^4^Laboratory of Microbiology, Hospital Professor Doutor Fernando Fonseca, Amadora, Portugal

**Keywords:** QepA4, quinolone resistance, genetic characterization, PaβN, WGS

## Abstract

A new QepA4 variant was detected in an O86:H28 ST156-*fimH38 Escherichia coli*, showing a multidrug-resistance phenotype. PAβN inhibition of *qepA4-*harboring transconjugant resulted in increase of nalidixic acid accumulation. The *qepA4* and *catA1* genes were clustered in a 26.0-kp contig matching an IncF-type plasmid, and containing a Tn21-type transposon with multiple mobile genetic elements. This QepA variant is worrisome because these determinants might facilitate the selection of higher-level resistance mutants, playing a role in the development of resistance, and/or confer higher-level resistance to fluoroquinolones in association with chromosomal mutations.

## Introduction

Quinolones are broad-spectrum antibiotics that have been used in medical practice for the treatment of severe or resistant infections (Kim and Hooper, 2014). This class of antibiotics is fully synthetic and used widely in both human and veterinary medicine.

Bacterial resistance to fluoroquinolones has emerged quickly and has conventionally been attributed to chromosomally encoded mechanisms that allow the alteration of quinolone targets (QRDR, quinolone resistance-determining regions): DNA gyrase and topoisomerase IV (Jacoby et al., 2014). However, the discovery of plasmid-borne determinants has increased the genetic background on the mechanisms of quinolone resistance, such as the plasmid-mediated fluoroquinolone resistance (PMQR) determinants.

The *qepA* gene is a PMQR gene encoding a 14-transmembrane-segment efflux pump of the major facilitator superfamily (MFS). Unlike other MFS efflux pumps that typically export various antimicrobial agents and dyes, QepA shows substrate specificity directed to the fluoroquinolones ciprofloxacin and norfloxacin (Jacoby et al., 2014; Rodríguez-Martínez et al., 2016).

QepA1 was described in *Escherichia coli* clinical isolates from Japan and Belgium in 2007 (Périchon et al., 2007; Yamane et al., 2007). Since then two new variants have been described: QepA2, in a CTX-M-15-producing *E. coli* isolated from urine and blood samples of a patient suffering from pyelonephritis, in France, in 2007 (Cattoir et al., 2008); and, more recently, QepA3, in an *Enterobacter aerogenes* isolated in 2011 from wound pus of a patient admitted at a Chinese hospital (Wang et al., 2015). These QepA2 and QepA3 variants differ from QepA1 by two (Ala99Gly and Val134Ile) and five (Ala235Glu, Pro274Leu, Trp318Cys, Met372Lys, and Ala445Thr) amino acids, respectively (Table 1).

**Table 1:** Comparison of amino acid substitutions, and epidemiology of first reports of clinical QepA-producing strains.

**PMQR gene**	**Amino acid at position no**.								**Strain^a^**	**Country^b^**	**References**
	**95**	**99**	**134**	**235**	**274**	**318**	**372**	**445**			
*qep*A1	Phe	Ala	Val	Ala	Pro	Trp	Met	Ala	*E. coli* (unknown)	Japan and Belgium (2007)	Périchon et al., 2007; Yamane et al., 2007
*qep*A2		Gly	Ile						*E. coli* (urine and blood)	France (2008)	Cattoir et al., 2008
*qep*A3				Glu	Leu	Cys	Lys	Thr	*E. coli* (blood, and sputum), *C. koseri* (sputum), *K. pneumoniae* (blood), *E. cloacae* (chest wound)	China (2015)	Wang et al., 2015
*qep*A4	Leu		Ile						*E. coli* (urine)	Portugal	This study

a *QepA-producing isolate, and respective human biological product of isolation (in parentheses), in the first report*.

b *Country that first reported the PMQR, and year (in parenthesis)*.

Antibiotic resistance genes are frequently associated to mobile genetic elements (MGE), such as insertion sequences (ISs), phages, transposons and plasmids, which enhance their ability to efficiently spread among different bacterial species (Stokes and Gillings, 2011). The occurrence of MGE harboring multiple antibiotic resistance genes is also frequent, and enables the development of bacterial multidrug-resistance (MDR), which may be responsible for therapeutic failures (Poirel et al., 2012; Kim and Hooper, 2014). Indeed, PMQRs are commonly described in isolates co-producing plasmid-mediated β-lactamases (PMAβ) and extended-spectrum β-lactamases (ESBL) (Jacoby et al., 2014).

In this study, we have identified and characterized the fourth variant of the QepA determinant-QepA4, which is responsible for the increased levels of resistance to clinically important quinolones. Furthermore, this is also, at our knowledge, the first description of the co-production of QepA and both the PMAβ CMY-2 and the penicillinase TEM-1. The study highlights the need of surveillance of this resistance mechanism and reinforces a more careful use of quinolones.

## Methods

### Antibiotic susceptibility testing and molecular characterization

Minimum inhibitory concentrations (MICs) of *E. coli* INSRA6015 isolated from a Portuguese healthcare facility were determined by microdilution and *E*-test methods according to European Committee on Antimicrobial Susceptibility Testing (EUCAST) guidelines (Table 2). Interpretation of the results was done according to the EUCAST epidemiological cut-off values (http://mic.eucast.org/Eucast2/). Detection and identification of β-lactamase- and PMQR-encoding genes, as well as the analysis of the quinolone-resistance-determining region (QRDR) was performed as previously described (Jones-Dias et al., 2013).

**Table 2:** MICs (mg/L) of antibiotics for the *E. coli* (Ec) strains: clinical INSRA6015, EcTOP10 (pBK-*qepA1*), and EcTOP10 (pBK-*qepA4*) transformants, and the recipient EcTOP10 (pBK-*qepA*^−^).

**Antibiotic**	**Ec INSRA6015 (*qepA4*, *bla*_TEM–1_, *bla*_CMY–2_)**	**EcTOP10 (pBK-*qepA^−^*)**	**EcTOP10 (pBK-*qepA1*)**	**EcTOP10 (pBK-*qepA4*)**
β**-LACTAMS**^a^				
Amoxicillin	1,024	2	4	4
Amoxicillin + CLA	128	4	4	4
Ticarcillin	>4,096	≤0.125	4	4
Piperacillin	128	≤0.015	1	1
Piperacillin + TAZ	4	≤0.015	≤0.015	≤0.015
Mecillinam	1	≤0.015	0.25	0.125
Cefuroxime	16	4	4	4
Ceftazidime	8	0.125	0.125	0.125
Ceftazidime + CLA	8	0.125	0.125	0.125
Ceftriaxone	8	0.03	0.03	0.03
Ceftriaxone + CLA	4	0.03	≤0.015	≤0.015
Cefotaxime	4	0.06	0.06	0.06
Cefotaxime + CLA	4	0.03	0.03	0.03
Cefoperazone	8	≤0.25	≤0.25	≤0.25
Cefepime	0.125	≤0.015	≤0.015	≤0.015
Cefoxitin	32	1	1	1
Aztreonam	4	0.06	0.06	0.06
Aztreonam + CLA	4	0.03	0.03	0.03
Imipenem	0.125	0.125	≤0.06	0.125
**OTHER CLASSES**^a^				
Kanamycin	2	≤0.125	≤0.125	≤0.125
Trimetoprim	>512	≤0.125	≤0.125	≤0.125
**QUINOLONES**^b^				
Nalidixic Acid	>256	≤0.015	1	0.75
Nalidixic Acid + PaβN	–	≤0.015	0.125	0.125
Ciprofloxacin	1024	≤0.002	0.016	0.007
Ciprofloxacin + PaβN	–	≤0.002	0.006	≤0.002
Enrofloxacin	>32	≤0.002	0.004	≤0.002
Enrofloxacin + PaβN	–	≤0.002	≤0.002	≤0.002
Gatifloxacin	>32	≤0.002	0.004	≤0.002
Gatifloxacin + PaβN	–	≤0.002	≤0.002	≤0.002
Levofloxacin	>32	0.004	0.008	0.004
Levofloxacin + PaβN	–	≤0.002	≤0.002	≤0.002
Moxifloxacin	>32	≤0.002	≤0.002	≤0.002
Moxifloxacin + PaβN	–	≤0.002	≤0.002	≤0.002
Norfloxacin	>256	0.016	0.38	0.094
Norfloxacin + PaβN	–	0.016	0.19	0.094
Ofloxacin	>32	0.008	0.016	0.012
Ofloxacin + PaβN	–	0.004	0.004	0.003

–*, Non determined*.

a *MICs determined by microdilution method*.

b *MICs determined by E-test*.

*PaβN, efflux pump inhibitor phenyl-arginine-β-naphthylamide at 50 μg/mL*.

### Gene transfer experiments

In order to characterize the QepA4 determinant, transformants were obtained by amplifying *qepA4* with primers qepA-F (5′–CGTTAAAGCATTCTTGTCCCGG–3′) and qepA-R (5′–ATGTCCGCCACGCTCCACG–3′), cloning it in the pBK-CMV phagemid vector (Stratagene), and transforming it into TOP10 OneShot chemically competent *E. coli* cells (Invitrogen). Susceptibility of transformants to an assortment of fluoroquinolones was tested alone and in the presence of 50 μg/mL of the efflux pump inhibitor phenyl-arginine-β-naphthylamide (PAβN) (Sigma-Aldrich) (Périchon et al., 2007), as mentioned above (Table 2). EcTOP10 (pBK-*qepA1*) strain was used as control.

### Genomic characterization of QepA4-producing *E. coli*

The QepA4-producing *E. coli* was characterized by whole-genome sequencing (WGS), as previously described (Manageiro et al., 2015). Briefly, genomic DNA was extracted using DNeasy Blood and Tissue Kit (Qiagen) and quantified using Qubit 1.0 Fluorometer (Invitrogen). The Nextera XT DNA Sample Preparation Kit (Illumina) was used to prepare sequencing libraries from 1 ng of genomic DNA according to the manufacturer's instructions. WGS was performed using 150 bp paired-end reads on a MiSeq (Illumina). Sequence reads were trimmed and filtered according to quality criteria, and *de novo* assembled into contigs by means of CLC Genomics Workbench 10.0 (QIAGEN). PathogenFinder 1.1, ResFinder 2.1, VirulenceFinder 1.4, SerotypeFinder 1.1, MLST 1.8, pMLST 1.4, and ISSaga were used to estimate the pathogenicity determinants, acquired antibiotic resistance genes, virulence factors, serotype, MLST, plasmid MLST, and insertion sequence regions, respectively in the genomes of PMQR-producing *E. coli* (Manageiro et al., 2015).

## Results and discussion

INSRA6015 showed non-susceptibility to fluoroquinolones (*E*-test method) and to third-generation cephalosporins, aztreonam, and cefoxitin (microdilution method); no synergy with clavulanate was detected. The *E. coli* isolate remained susceptible to piperacillin/tazobactam and imipenem (Table 2).

The β-lactamase-encoding genes *bla*_TEM–1_ and *bla*_CMY–2_, and the PMQR-encoding gene *qepA* were detected by molecular methods. Sequence analysis of the QepA variant showed amino acid substitutions Phe95Leu and Val134Ile, justifying the new name QepA4 (Table 1). Sequencing of QRDR revealed the presence of three amino acid substitutions in the correspondent proteins: Ser83Leu and Asp87Asn in GyrA subunit of DNA-gyrase, and Glu84Lys in the ParC subunit of topoisomerase-IV. These results are consistent with the detected high levels of resistance to fluoroquinolones (MIC >32 mg/L) (Table 2).

EcTOP10 (pBK-*qepA4*) strain revealed susceptibility to all fluoroquinolones and remaining antibiotics classes tested. In fact, susceptibility levels were similar to the ones showed by EcTOP10 (pBK-*qepA1*) strain, with exception of non-susceptibility to norfloxacin (0.094 vs. 0.38 mg/L, respectively). However, the *qepA4*-encoding transformant showed higher MIC values to nalidixic acid (≥6-fold), ciprofloxacin (≥2-fold) and norfloxacin (3-fold) than the *qepA*-negative strain. Moreover, the levels of susceptibility to nalidixic acid upon QepA with and without inhibition by PAβN were 3-fold higher for *qepA4*- and *qepA1-*encoding transformants (Table 2). These results show variability in the level of *qepA* expression, as previously discussed (Rodríguez-Martínez et al., 2016).

WGS allowed the characterization of the *qepA4* genetic background. The analysis yielded 91 contigs (from 203 to 358,909 bp), with a minimum 64-fold coverage. The draft genome contained a total assembly length of 4,770,076 bp, with a mean coverage of about 160-fold; the GC content was 50.9%. All *de novo* contigs were searched against the GenBank complete plasmids database using Megablast, with 14/91 contigs mapping against plasmid sequences therein deposited. Full details of these contigs are available in Table 3.

**Table 3:** Assembled contigs representing plasmids identified after BLASTn searched against the NCBI plasmid database.

**Contigs^a^**	**Resistance determinants**	**Strain name**	**Query cover (%)**	**Identity (%)**	**Accession number**
LLKU01000012		*Escherichia coli* strain FORC_031 plasmid pFORC31.1	100	100	NZ_CP013191.1
LLKU01000032	*catA1*-type	*Klebsiella pneumoniae* subsp. pneumoniae KPX plasmid pKPX-1 DNA	100	100	NC_021198.1
LLKU01000037		*Escherichia coli* strain CH613_eco plasmid unnamed2	98	100	NZ_CM007909.1
LLKU01000042, LKU01000051		*Salmonella enterica* subsp. enterica serovar Quebec str. S-1267 plasmid punamed2	100	98	NC_020278.2
LLKU01000044	***qepA4***	*Escherichia coli* strain 3A11 plasmid pHN3A11	100	99	NC_020278.2
		*Escherichia coli* JJ1887 plasmid pJJ1887-5			NZ_CP014320.1
LLKU01000049	*catA1*-type, *sul1, ΔdfrB4, mer* operon, ethidium bromide resistance protein	*Escherichia coli* strain CD306 plasmid pCD306	98	99	NZ_CP013832.1
LLKU01000055	*catA1*-type	*Escherichia coli* plasmid pH2291-144	100	100	NC_025139.1
LLKU01000058		*Salmonella enterica* subsp. *enterica* serovar Senftenberg NCTC10384, plasmid: 4			NZ_LN868946.1
		*Escherichia coli* strain 207 plasmid unnamed			NZ_CP019559.1
LLKU01000057, LKU01000060	*tet(B)*	*Salmonella enterica* subsp. *enterica* serovar Heidelberg strain N13-01290 plasmid pN13-01290_23	100	100	NZ_CP012931.1
		*Escherichia fergusonii* ATCC 35469 plasmid pEFER			NC_011743.1
LLKU01000068		*Escherichia coli* UMNF18 plasmid pUMNF18_87	99	99	NZ_AGTD01000003.1
LLKU01000069		*Citrobacter freundii* strain 705SK3 plasmid p705SK3_1	100	100	NZ_CP022152.1
		*Escherichia coli* strain EC1515 plasmid pEC1515-2			NZ_CP021846.1
LLKU01000073		*Escherichia coli* JJ1887 plasmid pJJ1887-5	100	100	NZ_CP014320.1

*Only the best BLASTn hit(s) reference plasmid sequence is shown (e value = 0.0, query cover ≥ 98% and identity ≥ 98%)*.

a *Contigs underlined includes the resistance determinants*.

**Figure 1:**
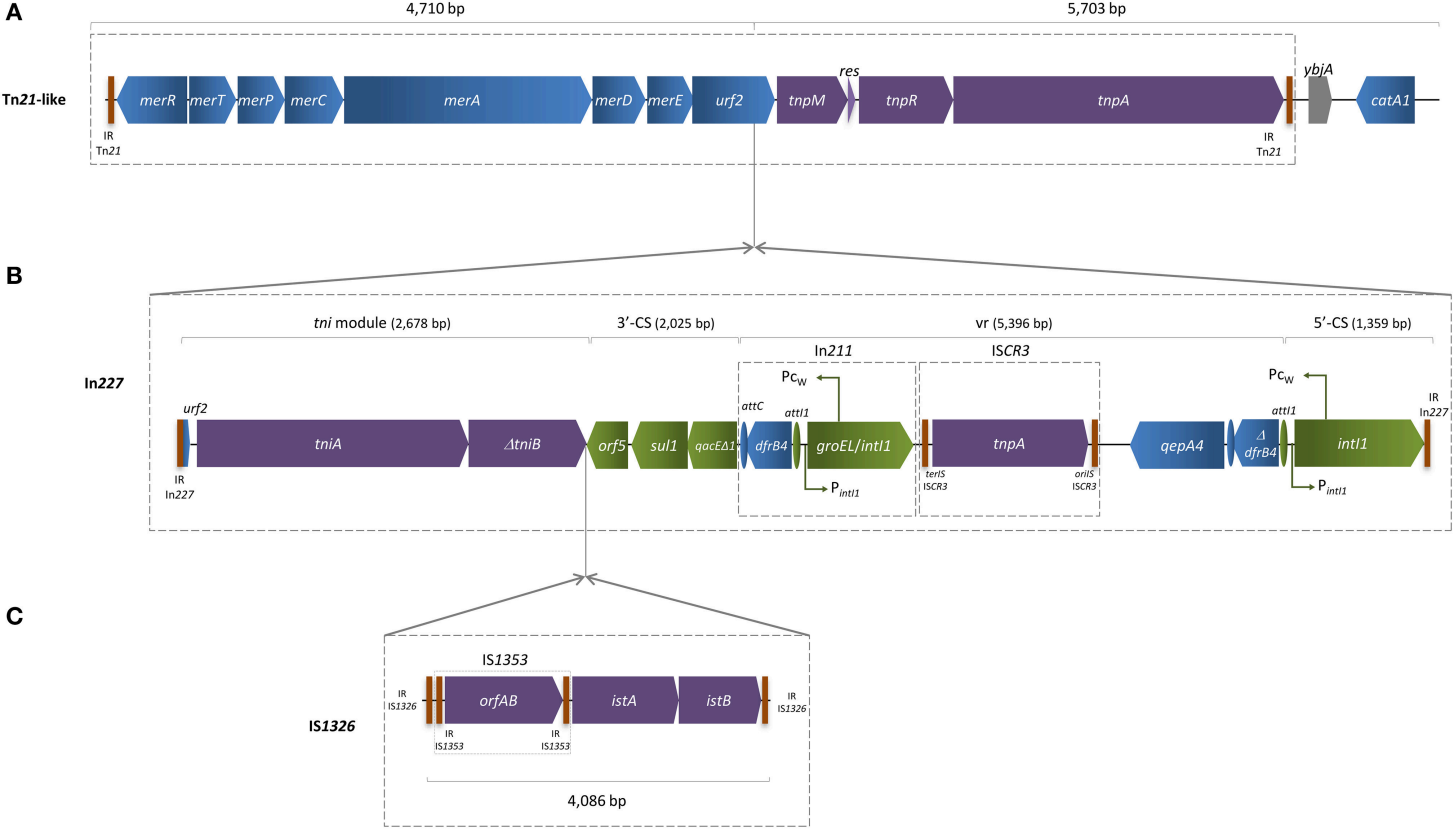
Schematic representation of *qepA4-*harboring contig assembled using LLKU01000032, LLKU01000034, LLKU01000049, and LLKU01000073, with mean coverage of 134-fold and 59.0% of GC content. **(A)** The Tn*21*-like transposon included the transposition (*tnp*) region [comprising the transposase (*tnpA*), the resolvase (*tnpR*), and the putative transposition regulator (*tnpM*)]; the resolution site (*res*); and the mercury resistance (*mer*) operon [with the regulatory genes *merR* and *merD*, the structural genes *merT, merP, merC*, and *merA*, and two unknown reading frames: *urf1* (also called *merE*) and *urf2*, downstream of *merD*]; **(B)** The *urf2* gene was interrupted by a complete In*227* integron, which variable region (vr) harbored a Δ *dfrB4* and a *qepA4* gene downstream an IS*CR3* element, flanked upstream by an In*211* integron; **(C)** Two ISs (IS*1353* inserted into IS*1326*) interrupted the *tniB* (transposition of the integron) gene. IR, flanking inverted repeats; *ori*IS and *ter*IS, origin and terminus of IS*CR* elements, respectively; vertical bars indicate IR of transposons (Tn), integrons (In) and insertion sequences (IS). Arrows indicate the direction of transcription of the various genes. Truncated genes are indicated by a *Δ* symbol.

The *qepA4* gene was found in a 25,957 bp length contig, which enclosed a composite mercury resistance Tn*21*-like transposon (Figure 1), showing 99% identity with previously described IncFII NR1 low-copy-number natural plasmid (Williams et al., 2006), over 80% length coverage. The region located between *urf2* and *tnpM* (Figure 1A) was interrupted by a complete In*227* (Figure 1B), which included IS*1353* inserted into IS*1326*, thus interrupting *tniB* gene (Figure 1C). The variable region harbored a Δ*dfrB4* and a *qepA4* gene downstream of an IS*CR3* element, which was flanked upstream by In*211*. Downstream of the Tn*21*-like transposition region, two acetyltransferase genes (*catA1, ybjA*) were detected. Unlike *qepA1* or *qepA3*, this *qepA4* gene was not genetically associated to the *rmtB* gene encoding a plasmid-mediated ribosomal methylase, which is consistent with susceptibility to aminoglycosides showed by INSRA6015. The remaining sequence of the contig matched with previously described *qepA*-harboring IncFII plasmids, such as pJJ1887-5 and pHN3A11, reported in the USA and China, respectively (Chen et al., 2014; Johnson et al., 2016), which correlates with PCR-based replicon results obtained (Carattoli et al., 2005). Horizontal transfer of the *qepA4* gene was not achieved either by bacterial conjugation or through the direct transformation of plasmid DNA (data not shown), suggesting the presence of a non-transferable plasmid.

Further analysis revealed the presence of genes conferring resistance to β-lactams (*bla*_TEM–1_, *bla*_CMY–2_), tetracycline [*tet(B)*], phenicol (*catA1-*type), sulphonamides (*sul1*) and trimethoprim (*dfrB4*-type). CMY-2- and TEM-1-encoding genes (LLKU01000025 and LLKU01000032 contigs, respectively), contrarily to the other genes found, were not detected within contigs that mapped against plasmid sequences (with ≥98% of query coverage and ≥98% of identity) (Table 3). According with the methodology used, the strain does not have other typeable plasmids.

Overall, 53 putative ORFs related to ISs were found within the INSRA6015 genome: 21 complete, 28 partial, and 4 uncategorized (including IS*1*, IS*1380*, IS*21*, IS*3ssgr*IS*3*, IS*As1*, and Tn*3* with 100% of similarity with those described in the ISsaga database. Three virulence factors (*iss, lpfA, gap*) were identified. Moreover, this O86:H28 *E. coli* isolate displayed a prediction of 93.4% for being a human pathogen, based on the probability scores assigned by PathogenFinder.

In addition, QepA4-producing *E. coli* presented a set of genetic features crucial to support their own successful dissemination (Figure 1), such as multiple antibiotic resistance genes carried by MGE, virulence factors and numerous other pathogenicity factors. These features enlarge bacteria ability for transboundary dissemination among bacteria from different environments (Stokes and Gillings, 2011; Caniça et al., 2015).

The QepA4-producing INSRA6015 *E. coli* isolate belonged to the ST156 lineage (UCC scheme) and ST119 (Pasteur scheme), and showed the *fimH38* allele upon *fimH* typing. This ST has been reported associated with different antibiotic-resistance genes, namely PMAβ and ESBL, NDM carbapenemases, and 16S rRNA methylases, both in clinical and colonizing human-associated *E. coli* isolates collected in different countries, and in water samples from Bangladesh (Corvec et al., 2010; Mushtaq et al., 2011; Pan et al., 2013; Sáez-López et al., 2014; Rashid et al., 2015). Indeed, the *qepA4* variant identified in this clinical isolate matched a partial QepA-type sequence (Accession Number LK934678) detected in a TEM-1 and CTX-M-15-producing *E. coli* strain recovered in a raw wastewater sample in Portugal, but from a different ST (ST443) (Varela et al., 2015).

The PMQR determinants confer low-level quinolone resistance that, in some cases, does not exceed the clinical breakpoint for susceptibility as demonstrated in this study and by others (Jacoby et al., 2014). However, its presence may facilitate higher-level resistance under selective pressure from antimicrobial agents at therapeutic levels, mostly due to chromosomal mechanisms, which makes infection by pathogens containing PMQR harder to treat (Poirel et al., 2012). Fortunately, the *qepA4* gene detected in this study was not in a genetic linkage to *rmtB* gene as has been demonstrated; this suggests that are no potential for selection of the QepA4 determinant by the use of aminoglycosides.

## Conclusion

In conclusion, *qepA4* was here first identified in an *rmtB*-negative clinical isolate, and genetically characterized within a composite mercury resistance Tn*21*-like transposon, harboring other different mobile genetic elements. This report represents an important finding about a plasmid-mediated resistance mechanism, which contributes with other quinolone resistance mechanisms to increase therapeutic failures, and understanding of this resistance in different reservoirs (Poirel et al., 2012; Yan et al., 2017).

## Data access

This draft genome has been deposited at DDBJ/EMBL/GenBank under the accession LLKU00000000. The version described in this paper is version LLKU01000000. The *qepA4* nucleotide sequence from this study was submitted to the GenBank Database with accession number KX686116.

## Author contributions

VM designed the study, performed molecular experiments, bioinformatics analysis, analyzed the data and wrote the manuscript. DF, DJ, and EF performed microbiological and molecular experiments, and analyzed the data. DS and LV performed Illumina genome sequencing experiments. LS acquired laboratory data. MC designed the study, wrote and reviewed the manuscript. All authors read and approved the final manuscript.

### Conflict of interest statement

The authors declare that the research was conducted in the absence of any commercial or financial relationships that could be construed as a potential conflict of interest.
